# Beneficial bacterial-*Auricularia cornea* interactions fostering growth enhancement identified from microbiota present in spent mushroom substrate

**DOI:** 10.3389/fmicb.2022.1006446

**Published:** 2022-10-10

**Authors:** Chitwadee Phithakrotchanakoon, Sermsiri Mayteeworakoon, Paopit Siriarchawatana, Supattra Kitikhun, Piyanun Harnpicharnchai, Supaporn Wansom, Lily Eurwilaichitr, Supawadee Ingsriswang

**Affiliations:** ^1^Microbial Systems and Computational Biology Research Team, Thailand Bioresource Research Center, National Center for Genetic Engineering and Biotechnology, National Science and Technology Development Agency, Pathum Thani, Thailand; ^2^National Energy Technology Center, National Science and Technology Development Agency, Pathum Thani, Thailand

**Keywords:** *Auricularia cornea*, growth-promoting bacteria, bacterial-fungal interactions, microbiota, co-cultivation, *Pseudonocardia*, proteomics, functional analysis

## Abstract

Complex dynamic bacterial-fungal interactions play key roles during mushroom growth, ranging from mutualism to antagonism. These interactions convey a large influence on mushroom’s mycelial and fruiting body formation during mushroom cultivation. In this study, high-throughput amplicon sequencing was conducted to investigate the structure of bacterial communities in spent mushroom substrates obtained from cultivation of two different groups of *Auricularia cornea* with (A) high yield and (B) low yield of fruiting body production. It was found that species richness and diversity of microbiota in group (A) samples were significantly higher than in group (B) samples. Among the identified 765 bacterial OTUs, 5 bacterial species found to exhibit high differential abundance between group (A) and group (B) were *Pseudonocardia mangrovi*, *Luteimonas composti*, *Paracoccus pantotrophus*, *Sphingobium jiangsuense*, and *Microvirga massiliensis*. The co-cultivation with selected bacterial strains showed that *A. cornea* TBRC 12900 co-cultivated with *P. mangrovi* TBRC-BCC 42794 promoted a high level of mycelial growth. Proteomics analysis was performed to elucidate the biological activities involved in the mutualistic association between *A. cornea* TBRC 12900 and *P. mangrovi* TBRC-BCC 42794. After co-cultivation of *A. cornea* TBRC 12900 and *P. mangrovi* TBRC-BCC 42794, 1,616 proteins were detected including 578 proteins of *A. cornea* origin and 1,038 proteins of *P. mangrovi* origin. Functional analysis and PPI network construction revealed that the high level of mycelial growth in the co-culture condition most likely resulted from concerted actions of (a) carbohydrate-active enzymes including hydrolases, glycosyltransferases, and carbohydrate esterases important for carbohydrate metabolism and cell wall generation/remodeling, (b) peptidases including cysteine-, metallo-, and serine-peptidases, (c) transporters including the ABC-type transporter superfamily, the FAT transporter family, and the VGP family, and (d) proteins with proposed roles in formation of metabolites that can act as growth-promoting molecules or those normally contain antimicrobial activity (e.g., indoles, terpenes, *β*-lactones, lanthipeptides, iturins, and ectoines). The findings will provide novel insights into bacterial-fungal interactions during mycelial growth and fruiting body formation. Our results can be utilized for the selection of growth-promoting bacteria to improve the cultivation process of *A. cornea* with a high production yield, thus conveying potentially high socio-economic impact to mushroom agriculture.

## Introduction

*Auricularia*, also known as wood ear mushroom, is the third most cultivated mushroom and accounts for approximately 17% of mushroom production worldwide after *Lentinula* (22%) and *Pleurotus* (19%) (Royse et al., 2017). The commercial cultivation of *Auricularia* mushrooms is mainly done in China, Indonesia, Malaysia, the Philippines, Thailand, and Vietnam (Royse et al., 2017). A fruiting body of *Auricularia* mushrooms is rich in several nutrients such as carbohydrates, proteins, dietary fibers, amino acids, and microelements that show beneficial properties including blood fat lowering, anti-tumor, anti-gastrointestinal cancer, and anti-oxidation activities (Bandara et al., 2019). Moreover, the fruiting body of *Auricularia polytricha* has been reported to be utilized as biosorbents for heavy metals such as Pb(II), Cd(II), Cu(II), and Cr(VI), which are beneficial for remediation of aqueous solution in industries (Zheng et al., 2014).

For commercial production of *Auricularia* mushrooms, *Auricularia* mycelia are normally inoculated in a bag containing substrates comprising sawdust as the main component supplemented with materials such as bran, straw, and gypsum. The spawn is allowed to run completely in 2 weeks. Then, the bags are cut to allow the fruiting body to appear in 8–10 days (Royse et al., 2017). Due to the economic importance of *Auricularia* mushroom production, many studies relating to the cultivation of *Auricularia* mushroom have recently been reported. For example, suitable formulations of substrates for high-yield cultivation of *Auricularia* were investigated (Abd Razak et al., 2013). The supplementation of some grass plants in the substrate was reported to provide higher efficiency (ratio of fruiting body obtained per weight of substrate used) in cultivation of *Auricularia polytricha* (Liang et al., 2019). Additionally, new species of *Scytalidium* causing slippery scar, which resulted in a huge loss in mushroom productivity, was identified (Peng et al., 2014).

Several studies have reported that certain bacteria can positively or negatively influence the growth of mycelium and the formation of fruiting body of many types of mushrooms (Rainey, 1991; Cho et al., 2003; Largeteau and Savoie, 2010; Oh et al., 2018; Stanojevic et al., 2019; Chen et al., 2022; Hua et al., 2022). For example, fluorescent *Pseudomonas* spp. induce fruiting body development of *Pleurotus ostreatus* (Cho et al., 2003). *Pseudomonas putida* promotes a high rate of radial hyphal extension of *Agaricus bisporus* (Rainey, 1991). *Bacillus* spp. indirectly benefit *A. bisporus* growth by reducing the growth of pathogens (Stanojevic et al., 2019). Finally, cell-free fermentation broth of *Bacillus cereus* Bac 1 enhances the growth speed of *P. eryngii* (Chen et al., 2022). On the other hand, some bacteria show negative effects on the growth of mushrooms. As reported, 10 bacterial strains inhibit the mycelial growth of *Tricholoma matsutake* (Oh et al., 2018). *Burkholderia gladioli* and *P. tolaasii* cause cavity disease and brown blotch in *A. bisporus*, respectively (Largeteau and Savoie, 2010). These studies show that complex bacterial-fungal interactions can play key roles during mushroom cultivation, and positive bacterial-fungal relationship can greatly enhance mycelial growth and fruiting body formation during the cultivation. The positive bacterial-fungal interactions can entail direct or indirect mechanisms. The direct mechanisms mostly increase availability of nutrients or growth-stimulating factors to the cells, whereas the indirect mechanisms include production of antibiotics, lytic enzymes, and other metabolites that aid in defense against undesirable pathogens (Mahmud et al., 2021; Chen et al., 2022; Upadhyay et al., 2022). The growth of both the mushroom and the mushroom growth-promoting bacteria can also be enhanced by available nutrients in certain environments such as those present in the mushroom spent substrate (Carrasco et al., 2018; Wu et al., 2020; Upadhyay and Chauhan, 2022). In addition, certain mushroom growth-promoting bacteria can also adapt and function in various environments, e.g., a range of substrates and temperature (Jemsi and Aryantha, 2017; Zhang et al., 2018).

One challenge currently facing mushroom farmers is that the fruiting body yields are sometimes unpredictable even when grown in a controlled environment. These inconsistent yields most likely stem from endogenous microorganisms that are present in the substrates during cultivation (Zhang et al., 2018). Therefore, investigation of the microbial community that can affect mushroom growth can be beneficial for enhancing the mushroom yield. We hypothesized that certain bacteria present in the mushroom substrate exhibit mutualistic relationship and can promote the growth of *A. cornea* during cultivation. Identification of these beneficial bacteria will provide great benefits for *A. cornea* farming. To date, employing a culture-independent approach coupled with high-throughput sequencing technology is useful for characterizing microbial communities since they are not relied on the capability of microorganisms to grow in the laboratory. The techniques have been used to explore the bacterial composition in the mushroom substrates and casing soil (Oh et al., 2016; Benucci et al., 2019; Ke et al., 2019; Yang et al., 2019; Carrasco et al., 2020; Chen et al., 2022; Vieira and Pecchia, 2022). However, there is limited information on the substrate-associated bacterial communities and their relationship to the growth of *Auricularia* species (Zhang et al., 2018). Therefore, in this study, we aimed to search for potential mushroom growth-promoting bacteria by comparison of bacterial communities in spent mushroom substrates obtained from high- and low-yield spent substrates *via* metagenomic approach. The co-cultivation was also conducted to detect the pair-wise symbiotic relationship of *A. cornea* with selected bacterial species, and the growth-promoting bacteria for cultivation of *A. cornea* were reported. Then, we further explored the possible molecular mechanisms underlying bacterial-fungal interaction during co-cultivation *via* proteomic and bioinformatic approaches.

## Materials and methods

### Cultivation conditions of *Auricularia cornea*

Methods for preparation of mushroom substrate and *A. cornea* cultivation were adapted from studies of Zhang et al. and Thongklang et al. (Zhang et al., 2018; Thongklang et al., 2020). The mushroom substrate contained sawdust (92.55%), rice bran (5%), lime (1.25%), gypsum (0.5%), Epsom salts (0.2%) and rice flour (0.5%). All components were mixed. The mixture (1 kg) was then put in a plastic bag (6.5 × 12.5 inch) and steamed at 100°C for 6 h. After cooling to room temperature, 10–15 grains of millet spawn of *A. cornea* were inoculated and allowed to grow until the bags were full of hyphae. Then, the bag was cut to allow the fruiting body to develop. Houses for spawn incubation and fruiting production were at 25–32°C with 80% humidity. The fruiting body was collected during the period of cultivation and the weights of the collected fruiting body were quantified.

### Collection of the spent mushroom substrate samples and microbial DNA extraction

Based on the weights of the collected fruiting body (Supplementary Table S1), the spent mushroom substrate samples were divided into 2 groups, namely group A (high production yield) and group B (low production yield). Group A included 5 bags of spent mushroom substrate providing >200 g per bag of fruiting body and group B included 5 bags of spent mushroom substrate providing <200 g per bag of fruiting body. The spent mushroom substrate samples from each cultivation bag were mixed thoroughly before microbial DNA extraction. Total microbial DNA was extracted from 250 mg of each spent mushroom substrate sample in duplication by using the DNeasy PowerSoil Pro Kit (Qiagen) following the manufacturer’s instructions. The extracted DNA samples were visualized on 0.8% agarose gel. DNA concentration and purity were measured using the NanoDrop™ One/OneC Microvolume UV–Vis Spectrophotometer (ThermoFisher Scientific). All DNA samples were kept at −80°C, prior to DNA sequencing.

### Library preparation and sequencing

To determine bacterial communities, the hypervariable V3-V4 region of the bacterial 16S rRNA gene was amplified using primers 341F (5′CCTAYGGGRBGCASCAG3′) and 806R (5’GGACTACNNGGGTATCTAAT3’) with unique barcodes. All PCR reactions were carried out with Phusion® High-Fidelity PCR Master Mix (New England Biolabs). The PCR products were mixed at the same volume with 1X loading buffer containing SYBR green and analyzed on 2% agarose gel electrophoresis for visualization. Bright bands of approximately 400 bp were purified with QIAquick Gel Extraction Kit (Qiagen) and quantified *via* Qubit® 2.0 Fluorometer (ThermoFisher Scientific). Then, the purified PCR products were mixed in equidensity ratios and purified again. Sequencing libraries were generated with the NEBNext® Ultra™ DNA Library Prep Kit for Illumina® and quantified *via* Qubit® 2.0 Fluorometer. The sequencing was performed using Illumina platform with paired-end. Low-quality reads, adapters, and primers were removed from the raw sequencing results and paired-end sequences were assembled. The sample sequencing and data cleaning processes were proceeded by Novogene company.

### Analysis of microbial community

The partial sequences of the 16S rRNA gene (V3-V4 region) were used for Operational Taxonomic Units (OTUs) identification. Kraken 2 was used for assigning taxonomic labels to each OTU with k-mer length = 35 and confidence = 0.05 (Wood et al., 2019). Blast analysis against the 16S rRNA database of NCBI (downloaded on 18 November 2019) at >97% identity and *e*-value <1*e*-10 was additionally used for species identification. Subsequently, alpha diversity indices including Chao 1, Shannon, and Simpson were calculated. Beta diversity was calculated by Bray-Curtis method and plotted with Non-metric Multi-dimensional Scaling (NMDS) using metaMDS and ordiellipse functions in package vegan in R version 2.6–2 (Oksanen et al., 2022). Analysis of similarities (ANOSIM) between groups was conducted for the statistical test. To identify the OTUs that showed differential abundance between group A and group B samples, the number of reads for each OTU present in each sample was processed by DESeq2 package (version 1.26.0) (Love et al., 2014) and metagenomeSeq (version 1.28.2) (Paulson et al., 2013). In details, DESeq (differential gene expression analysis based on the negative binomial distribution) function in DESeq2 package and zero-inflated Gaussian model using cumulative sum scaling (CSS) normalized data in metagenomeSeq package were separately conducted to compare the differential abundance of species in group A and group B samples. The *p*-values from both methods were adjusted with Benjamini and Hochberg method to reduce the false discovery rate (Benjamini and Hochberg, 1995). Then, the names of differential species were identified by their consensus results. The overall workflow for differential species identification is shown in Supplementary Figure S1.

### Co-cultivation of bacteria with *Auricularia cornea*

Five bacterial strains including *Pseudonocardia mangrovi* TBRC-BCC 42794*, Pseudonocardia antitumoralis* TBRC 5646, *Pseudonocardia antitumoralis* TBRC-BCC 21728, *Sphingobium yanoikuyae* TBRC 5787, and *Microvirga lotononidis* TBRC 5398 were obtained from Thailand Bioresource Research Center (TBRC). The bacterial strains were pre-cultivated on nutrient agar (NA) at 25°C for 10 days while *A. cornea* (TBRC 12900) was pre-cultivated on potato dextrose agar (PDA) at 25°C for 7 days. The co-cultivation was done on potato dextrose agar (PDA) media supplemented with 10 g/l peptone in a 15 × 90 mm Petri dish. *A. cornea* mycelia with 5 mm diameter were placed in the middle of the agar media and each of the bacterial strains was line-streaked for 20 mm on one side of the mycelial piece (25 mm away from the center of agar media). Line-streak with sterilized distilled water was used instead of bacteria for negative control. The co-cultivated plates were then incubated at 25°C. The lengths of *A. cornea* mycelia away from the center of the colony in both directions (the side facing towards and away from bacterial streaked line) were measured after incubation for 3, 5, 7, and 10 days. The experiment was done in 5 replications. The difference between mycelial lengths facing towards and away from the bacterial colony was used to indicate the effect of co-cultivation of each bacterial strain with *A. cornea* TBRC 12900 when *L_d_* = *L_t_* – *L_a_*, where *L_d_* is the difference between mycelial lengths facing towards and away from the bacterial colony, *L_t_* is the mycelial length facing towards the bacterial colony, and *L_a_* is the mycelial length facing away from the bacterial colony. A high value of *L_d_* should indicate that the presence of the bacteria in the co-culture helped promote *A. cornea* mycelial growth. A linear regression line between differential mycelial lengths and days of cultivation was then plotted for the co-cultivation between *A. cornea* TBRC 12900 and each of the bacterial strains tested.

### Protein extraction and digestion for gel-free proteomic analysis

After *A. cornea* TBRC 12900 was co-cultivated with *P. mangrovi* TBRC-BCC 42794 at 25°C for 7 days, 20 mm х 5 mm pieces of media agar located between *A. cornea* mycelia and *P. mangrovi* cells were cut and placed in 1.5 ml tubes. Pieces of media agar with the same dimension were also obtained from single-culture plates containing culture of *A. cornea* TBRC 12900 or *P. mangrovi* TBRC-BCC 42794. The media agar pieces were lysed by lysis buffer solution (0.5% Triton X-100, 10 mM DTT, 10 mM NaCl in 50 mM HEPES-KOH pH 8.0), incubated at 25°C, 800 rpm for 60 min, and centrifuged at 20,000 х g for 10 min at 4°C. Proteins extracted were precipitated with fresh cold acetone and stored at −20°C overnight. After precipitation, the protein pellet was reconstituted in 0.25% RapidGest SF (Waters, United Kingdom)/10 mM Ammonium bicarbonate (AMBIC). The protein concentrations were determined by the Bradford Reagent assay kit (Sigma, United States), using bovine serum albumin as the standard. The total protein amount of 20 μg was subjected to trypsin digestion. Reduction of the sulfhydryl bonds by using 10 mM DTT in 10 mM AMBIC at 62°C for 20 min and alkylation of sulfhydryl was performed at room temperature for 25 min in the dark. The solution was cleaned up by the Desalting column (Thermo Scientific, United States). The flow-through solution was enzymatically digested by trypsin (Thermo Scientific, LT) at a ratio of 1:50 (enzyme: protein) and incubated at 37°C overnight. The digested peptides were reconstituted in 0.1% formic acid and transferred to a TruView LCMS vial (Waters, United Kingdom).

### LC–MS/MS proteomics analysis

A total of 1.0 μg peptides were subjected to LC–MS/MS. The spectrum data was collected in a positive mode on a sciex triple TOF-6600+ mass spectrometer (ABSCIEX, De) combined with a nano-liquid chromatography (LC) system (Thermo Scientific, United States) and a nano analytical column (75 μm i.d. x 15 cm, packed d with Acclaim PepMap™ C18) (Thermo Scientific, De). LC conditions were the following: mobile phase A and B were used. Mobile phase A composed of 0.05% Trifluoroacetic acid (TFA) and mobile phase B composed of 80% acetonitrile/ 0.04% TFA. The samples loaded onto nano analytical column were first separated using a linear gradient of 3–35% B for 95 min with the nano-LC system at a constant flow rate of 300 ml/min. The analytical column was regenerated at 90% B for 10 min and re-equilibrated at 5% B for 15 min. The eluted peptides were analyzed LC–MS/MS. The MS acquisition time was set from gradient time zero to 120 min, and the MS1 spectra were collected in the mass range of 400 to 1,500 m/z with 250 milliseconds in “high sensitivity” mode. Further fragmentation of each MS1 spectrum occurred with a maximum of 30 precursors per cycle. Switch criteria used were the following: charge of 2+ to 5+, 500 cps intensity threshold, and dynamic exclusion for 15 s.

The raw MS-spectra resulting (.wiff) files were converted into mzML files using MSconvert and then analyzed using a trans proteomic pipeline (TPP). The MS spectra were searched against the protein database consisting of proteins from *Pseudonocardia* and *A. cornea* using Comet. The comet search parameters included static modifications of cysteine (+57.021464 Da), peptide mass tolerance of 20 ppm, fragment bin tolerance of 1.005, and fragment bin offset of 0.4. Peptide-spectrum matches were validated using PeptideProphet and iProphet, and protein assignments were then performed using ProteinProphet. Label-free quantification of the proteins was performed using the StPeter with a minimum probability threshold of 0.95.

### Functional annotation of proteins

Genome sequences of *Pseudonocardia oroxyli*, *Pseudonocardia dioxanivorans*, *Pseudonocardia kujensis*, and *A*. *cornea* were analyzed using antiSMASH6.0 (Blin et al., 2021) for biosynthetic gene clusters (BGCs) identification. Biosynthetic class was assigned to the identified proteins by searching against the BGCs in the genome of *P. oroxyli*, *P. dioxanivorans*, *P. kujensis*, and *A. cornea*. Carbohydrate-active enzymes were annotated using the CAZymes database^1^ and dbCAN2^2^ (Zhang et al., 2018; Drula et al., 2022). Peptidase families and transporter families were assigned using BLAST 2.12.0 against the MEROPS 12.1 database^3^ and Transporter Classification database (TCDB^4^), respectively, using thresholding with *e*-value <1*e*-10 (Saier et al., 2006; Rawlings et al., 2018). Clusters of orthologous genes (COG) were classified using NCBI’s conserved domains search against the COG database V1.0^5^ using thresholding with *e*-value <1*e*-10 (Tatusov et al., 2000). Subcellular localization of the identified proteins was predicted using BUSCA^6^ (Savojardo et al., 2018).

### Construction of protein–protein interaction network

PFAM domain of each protein was assigned using interProScan for the domain-domain interaction (DDI) identification. Protein–Protein interaction (PPI) between *A. cornea* and *P. mangrovi* was inferred based on the associated DDI pairs using PPIDomainMiner,^7^ Gold category, and STRING database^8^ (von Mering et al., 2003; Alborzi et al., 2021). The DDI pairs were constructed to PPI networks and then were analyzed using Cytoscape (Shannon et al., 2003).

## Results

### Bacterial community of spent mushroom substrate

In this study, 10 samples of the spent mushroom substrate were divided into 2 groups, namely group A (high yield, >200 g fruiting body) and group B (low yield, <200 g fruiting body), with 5 biological repeats in each group (Supplementary Table S1). The 2 groups showed significantly different weights (*p*-value <0.05). The bacterial communities of spent mushroom substrate samples were analyzed by high-throughput sequencing with the hypervariable V3-V4 region of the bacterial 16S rRNA gene. A total of 977,115 raw reads were obtained from the 10 samples. After quality filtering, a total of 965,183 qualified reads were retained. Next, chimeric sequences were filtrated, resulting in the 755,522 reads remaining. The number of reads across all samples ranged from 67,794 to 84,315 (Supplementary Table S2). The average length (bp) of the sequences was from 423 to 427. The sequences from all samples were annotated into 765 OTUs with 97% similarity as the threshold. An average number of 722 and 627 OTUs were observed from the samples in group A and group B, respectively (Supplementary Table S2). The rarefaction curves indicated that the number of OTUs reached a plateau for all samples (Supplementary Figure S2).

**Figure 1:**
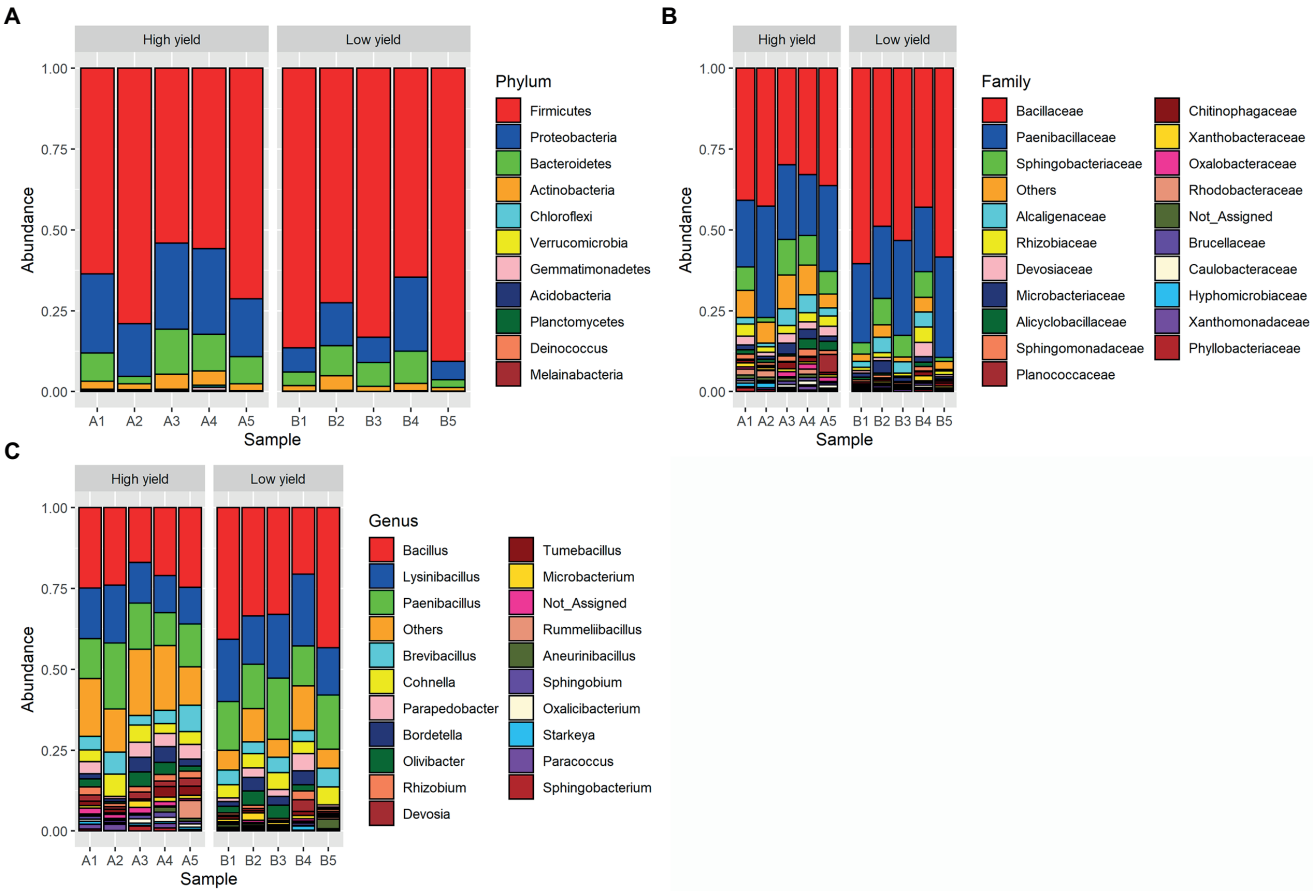
Taxonomic distribution of bacterial OTUs identified from group A (high yield) and group B (low yield) of spent substrates. The panels shown are **(A)** distribution at phylum level, **(B)** distribution at family level, and **(C)** distribution at genus level.

A total of 11 phyla, 31 classes, 64 orders, 124 families, and 329 genera of bacteria were detected from the spent substrate samples with the Kraken 2 taxonomic sequence classification tool. The taxonomic distribution at the phylum level is shown in Figure 1. Based on relative abundance, the most 4 abundant phyla in all samples were Firmicutes, Proteobacteria, Bacteroidetes, and Actinobacteria, respectively. Proteobacteria, Bacteroidetes and Actinobacteria were more abundant in group A (22.3, 8.9, and 3.1% respectively) than group B (11.4, 6.6, and 2.2%). Even though Firmicutes constituted the most abundant phyla in the spent substrate of both groups, they were more abundant in group B (79.5%) than in group A (64.7%). Using DESeq2, Firmicutes and Actinobacteria showed significant difference in abundance (value of *p* <0.05) between the 2 groups. Bacillaceae was the most dominant bacterial family found in both groups, followed by Paenbacillaceae and Sphingobacteriaceae, respectively. At the genus level, *Bacillus* was a dominant genus in both groups, followed by *Lysinbacillus*, and *Paenbacillus*. However, the percentages of *Bacillus*, *Lysinbacillus*, and *Paenbacillus* were lower in group A (22.3, 13.8, and 14.1%, respectively) compared to group B (34.2, 18.1, and 15.3%). On the other hand, the percentages of most other bacterial species are higher in group A than in group B.

**Figure 2:**
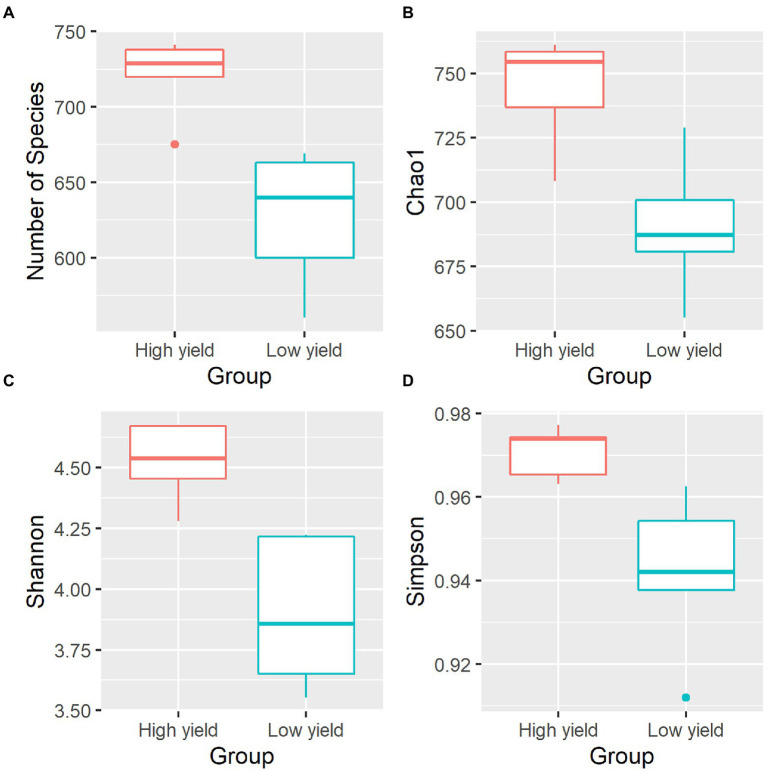
Alpha diversity indices of the bacterial community in spent mushroom substrate samples in group A and group B. All indices are plotted for samples in group A (red) and group B (cyan). The line inside the box represents the median. Statistical analysis shows significant differences of samples in group A and group B for all indices (pObserved = 0.006272, pChao 1 = 0.008897, pShannon = 0.007275, pSimpson = 0.025268). The panels shown are **(A)** the numbers of species identified, **(B)** Chao1 diversity index, **(C)** Shannon diversity index, and **(D)** Simpson diversity index.

To compare the microbial diversity in group A and group B samples, species richness and 3 different alpha diversity indices including Chao 1, Shannon, and Simpson were calculated based on the OTUs data at species level (Figure 2; Supplementary Table S2). By statistical analysis, all the indices revealed significant differences in the diversity between samples in group A and group B. As shown in Figure 2, the average species richness of samples in group A was 722, which was higher than that of group B with 627 species richness (pObserved = 0.006272). Similarly, Chao 1, Shannon, and Simpson indices were significantly higher in group A compared to group B (pChao 1 = 0.008897, pShannon = 0.007275, pSimpson = 0.025268). The results suggested that group A contained a significantly higher level of species diversity than group B.

**Figure 3:**
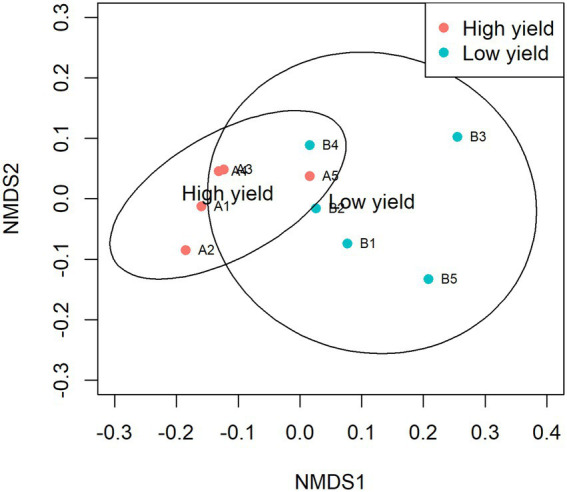
Non-metric multidimensional scaling (NMDS) of samples in group A and group B. The dissimilarity of 10 samples was calculated by Bray-Curtis and plotted with NMDS with 95% confidence interval. Samples in group A are presented as red circles and samples in group B are presented as cyan circles. The groups show significant differences in similarity analyzed by ANOSIM (pANOSIM = 0.031).

NMDS was plotted to estimate dissimilarities in the bacterial compositions between samples in group A and group B. Samples with more similarity to one another are ordinated closer together. As shown in Figure 3, samples A1, A2, A3, and A4 were ordinated closely together in the left and separately ordinated from samples in group B. Statistical analysis by ANOSIM confirmed the significant differences in the similarity between samples in group A and group B (pANOSIM = 0.031).

### Bacterial species with differential abundance between group A and group B

**Figure 4:**
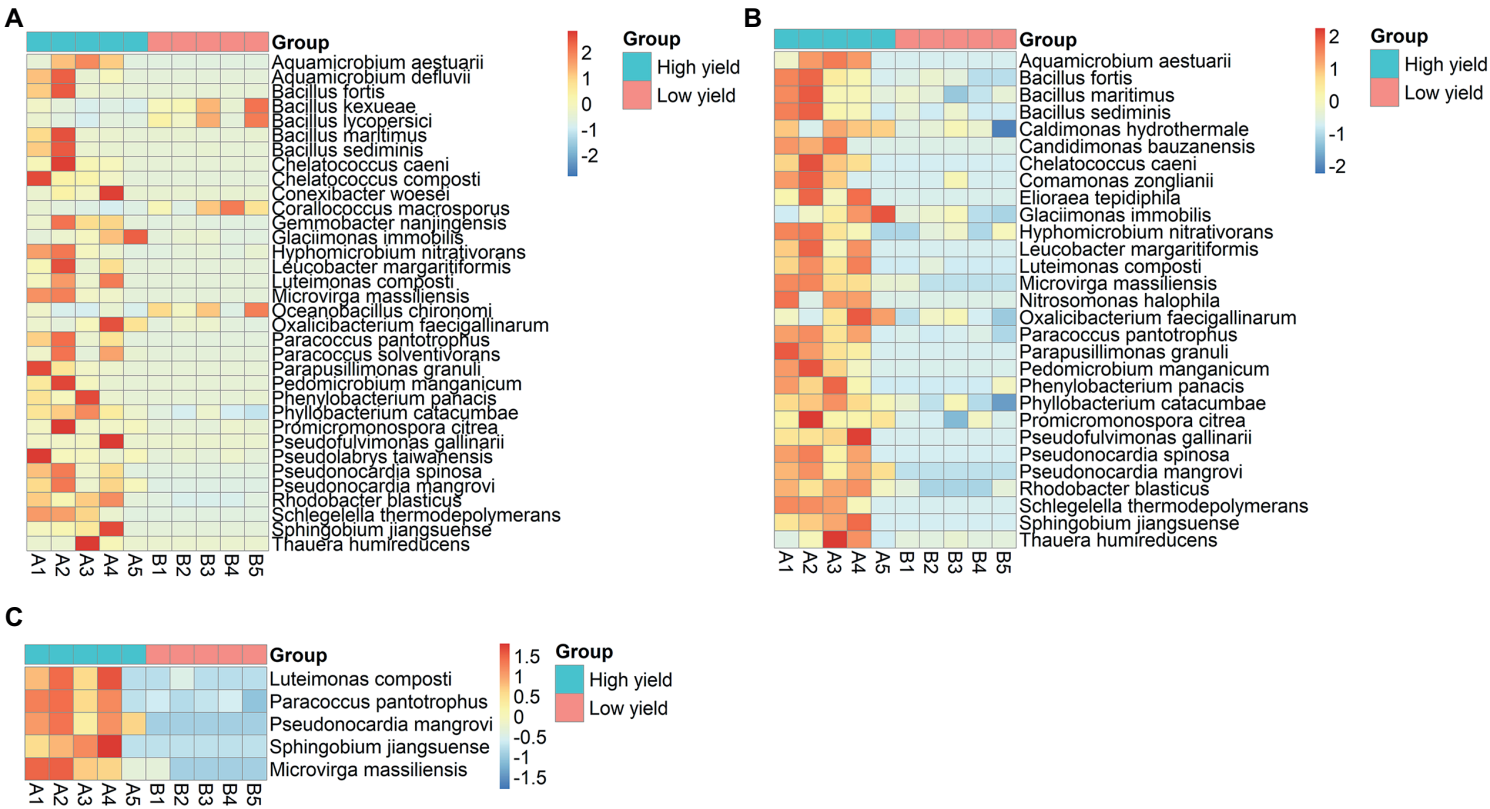
Heat-map demonstrating differentially abundant species between group A and group B analyzed by **(A)** DESeq2, **(B)** metagenomeSeq, and **(C)** merged results from both methods showing 5 species with the highest degrees of differential abundance. The row represents species, and the column represents the samples. The abundance of species corresponds to the color scale of the heat map.

According to the non-chimeric 755,522 reads, 386,253 reads could be identified to species level. Next, to identify the species with differential abundance between group A and group B, the abundances of bacterial species in both groups were analyzed using DESeq2 and metagenomeSeq. The result from DESeq2 revealed 34 differential species (*p*-value <0.05) (Figure 4A; Supplementary Table S3). Most of them showed higher abundance in group A than in group B, except for 4 species including *Corallococcus macrosporus*, *Oceanobacillus chironomi*, *Bacillus kexueae*, and *Bacillus lycopersici* that showed higher abundance in group B than in group A. When metagenomeSeq tool was employed, 29 differential species were significantly identified (*p*-value <0.05). All of them were higher abundance in group A than in group B (Figure 4B; Supplementary Table S3). After integrating the consensus-based results from the 2 methods, 24 species were assigned as the differential species and 5 species including *Luteimonas composti*, *Paracoccus pantotrophus*, *Pseudonocardia mangrovi*, *Sphingobium jiangsuense*, and *Microvirga massiliensis* showed the highest degrees of differential abundance (*p*-value <0.01) (Figure 4C and Supplementary Table S3).

### Assessment of growth-promoting bacteria in co-cultivation with *Auricularia cornea*

**Figure 5:**
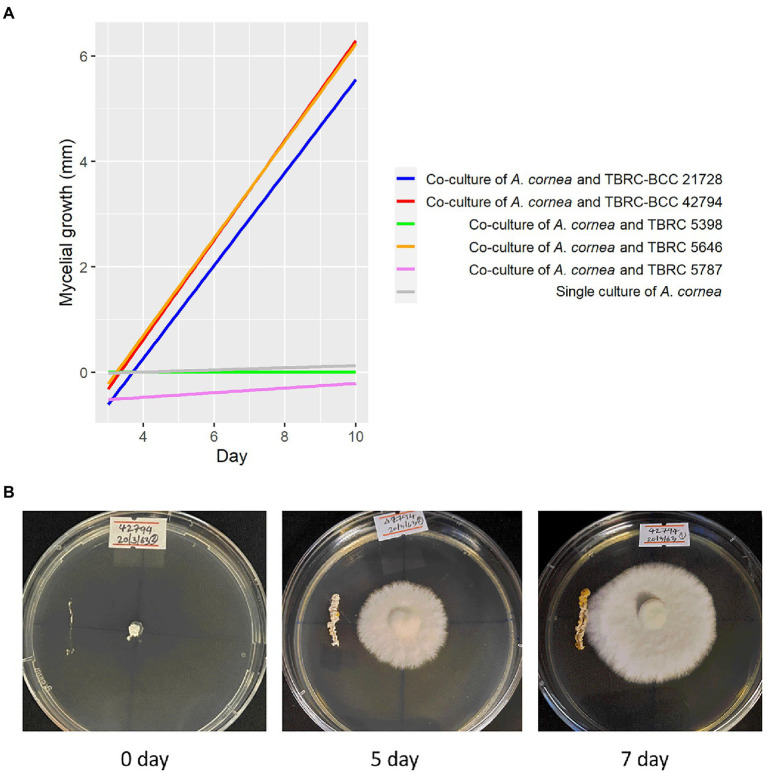
Effects of bacteria on the growth of *A. cornea* in the co-culture conditions on solid agar medium. **(A)** Linear regression lines showing *A. cornea* mycelial growth in the co-culture conditions are displayed as differences between mycelial length on the side towards the bacterial colony and on the side away from the bacterial colony in the graph. In the co-culture conditions, *A. cornea* TBRC 12900 was co-cultured with *P. mangrovi* TBRC-BCC 42794 (red line), *P. antitumoralis* TBRC-BCC 21728 (blue line), *M. lotononidis* TBRC 5398 (green line), *P. antitumoralis* TBRC 5646 (orange line), or *S. yanoikuyae* TBRC 5787 (pink line). The mycelial growth in the single culture of *A. cornea* (gray line) was used as a negative control. **(B)** Co-culture of *A. cornea* TBRC 12900 and *P. mangrovi* TBRC-BCC 42794 resulted in elevated mycelial growth on agar medium near the *P. mangrove* line streak (shown on day 5 and day 7 after the co-cultivation).

Bacterial strains that are available at Thailand Bioresource Research Center (TBRC) and belong to the same genera displaying high degrees of differential abundance identified in the spent mushroom substrate were selected to investigate their effects on the mycelial growth of *A. cornea*. They included 3 strains of *Pseudonocardia* (*P. mangrovi* TBRC-BCC 42794, *P. antitumoralis* TBRC 5646, and *P. antitumoralis* TBRC-BCC 21728), 1 strain of *Sphingobium yanoikuyae* TBRC 5787, and 1 strain of *Microvirga lotononidis* TBRC 5398. The effects of individual co-cultivation of these bacterial strains with *A. cornea* on solid media were observed during 10-day incubation by measuring the mycelial length of *A. cornea*. The lengths of *A. cornea* mycelia measuring from the center to the edge of the mycelia in the opposite directions (towards and away from the bacterial streaked line) were measured. The bacterial strains that promote the mycelial growth of *A. cornea* should result in a higher length of mycelia on the side towards the bacterial colony than on the side away from the bacterial colony. Thus, this difference in mycelial lengths facing towards and away from the bacterial colony can be used to indicate the effect of co-cultivation of each bacterial strain with *A. cornea* TBRC 12900. Its positive value would indicate the positive effect exerted by the bacterial colony in promoting mycelial growth of *A. cornea* TBRC 12900. When *A. cornea* was co-cultivated with each of the *Pseudonocardia* strains, the mycelial growth of *A. cornea* was significantly higher than that of control (without bacterial streaked line) (*p*-value <0.05), while no significant effect was observed from *S. yanoikuyae* and *M. lotononidis* (Figure 5).

### Proteomic analysis of interactions between *A. cornea* and *P. mangrovi*

As the co-culture between *A. cornea* TBRC 12900 and *P. mangrovi* TBRC-BCC 42794 resulted in the highest growth rate of *A. cornea*’s mycelium, the co-cultivation between these two organisms was investigated in more details. Proteomic analyses of the co-culture and single-culture of *A. cornea* TBRC 12900 or *P. mangrovi* TBRC-BCC 42794 were performed by liquid chromatography with tandem mass spectrometry to identify expressed proteins in the co-culture that most likely enhance the growth of *A. cornea*’s mycelium. From the co-culture, proteomic analysis successfully identified 1,038 and 578 proteins of *P. mangrovi* and *A. cornea* origins, respectively. In addition, 1,088 proteins and 585 proteins were identified from the single culture of *P. mangrovi* TBRC-BCC 42794 and single culture of *A. cornea* TBRC 12900, respectively. These proteins were then compared with the proteins in the Carbohydrate Active Enzyme (CAZy) database. The predicted extracellular proteins in each condition were identified using BUSCA. The identified proteins from the co-culture and single conditions were also compared to biosynthetic proteins annotated as components of biosynthetic gene clusters (BGCs). The presence of peptidases was detected with MEROPS. In addition, transporter members were classified using TCDB. Table 1 lists the number of CAZymes, peptidases, biosynthetic proteins, transporters, and extracellular proteins identified. It can be seen that the number of enzymes, biosynthetic proteins, and extracellular proteins (proteins with a signal peptide) from *A. cornea* increased in the co-culture condition compared to the single-culture condition.

**Table 1:** Number of proteins identified in the single-cultures and co-cultures of *Auricularia cornea* and *Pseudonocardia mangrovi.*

	**Single culture (*P. mangrovi*)**	**Single culture (*A. cornea*)**	**Co-culture (*P. mangrovi*)**	**Co-culture (*A. cornea*)**
Total Protein	1,088	585	1,038	578
CAZy	49	23	49	31
MEROPS	57	63	52	60
TCDB	156	84	137	82
Protein in BGC	27	8	26	10
Extracellular	40	73	34	76

Enzymes that are involved in the synthesis, breakdown, and binding of complex carbohydrates (e.g., disaccharides, oligosaccharides, polysaccharides, and glycoconjugates) should be vital for the cellular growth and development of an organism. These enzymes should be highly active during mycelial growth. The list of Carbohydrate-Active enzymes (CAZy) identified by dbCAN2 (Table 2) showed that the numbers of auxiliary activity (AA) enzymes and carbohydrate esterases (CE) from both *A. cornea* TBRC 12900 and *P. mangrovi* TBRC-BCC 42794 increased in the co-culture condition. Moreover, the numbers of enzymes with carbohydrate-binding modules (CBM) and glycosyltransferases (GT) identified from *A. cornea* TBRC 12900 also increased in the co-culture condition. The enzymes from *A. cornea* TBRC 12900 that increased during the co-culture included many important families including AA3, AA9, and AA14 from the auxiliary activity family, CBM1 and CBM42 from the carbohydrate-binding module family, CE15 and CE16 from the carbohydrate esterase family, GH2, GH28, GH38, GH43, and GH152 from the glycosyl hydrolase family, and GT2, GT20, GT35, and GT39 family from the glycosyltransferase family (Supplementary Table S4).

**Table 2:** Number of CAZy enzymes identified from the single- and co-culture conditions by dbCAN2.

**CAZymes family**	**Single culture (*P. mangrovi*)**	**Single culture (*A. cornea*)**	**Co-culture (*P. mangrovi*)**	**Co-culture (*A. cornea*)**	**Family description**
AA	2	0	4	5	Auxiliary activity
CBM	3	3	0	4	Carbohydrate binding modules
CE	4	2	5	4	Carbohydrate esterases
GH	12	13	12	13	Glycoside hydrolases
GT	29	7	29	10	Glycosyltransferases
PL	0	2	0	1	Polysaccharide lyases

As peptidases are a particular class of enzymes that provide nitrogen and amino acids to support growth and colonization of the fungi as well as involve in cellular processes such as generation of signaling molecules, the change in number of peptidases during the co-cultivation was further examined by comparing of protein-coding genes against the MEROPS database. As seen in Table 3, in the co-culture condition, a total number of 52 and 60 peptidases were identified as those from *P. mangrovi* and *A. cornea*, respectively. These peptidases are further grouped into aspartic-, cysteine-, metallo-, serine-, and threonine-peptidases based on their catalytic type. The most abundant peptidases identified from the co-culture are serine peptidases. Even though the total number of peptidases of *A. cornea* seems to decrease slightly in the co-culture condition, there is an increase in the number of serine peptidases of *A.cornea* in the co-culture condition. Interestingly, aspartic and threonine peptidases were uniquely found only from *A.cornea* and not from *P. mangrovi*. The most abundant peptidases of *A. cornea* found in both single- and co-culture included Copia transposon peptidases (family A11) of the aspartic peptidase family and prolyl oligopeptidases (family S09) from the serine peptidase family (Supplementary Table S5). The numbers of proteins in both types of peptidases (A11, S09) increased in co-culture condition. From *P. mangrovi*, the most abundant peptidases identified from both single- and co-culture included three families of serine peptidases including aminopeptidase peptidases (family S33), prolyl oligopeptidases (family S09), and signal peptide peptidase A (family S49). The S49 family was found only from *P. mangrovi* and not from *A. cornea*.

**Table 3:** Number of peptidases identified with MEROPS 12.1 database.

**MEROP Class**	**Single culture (*P. mangrovi*)**	**Single culture (*A. cornea*)**	**Co-culture (*P. mangrovi*)**	**Co-culture (*A. cornea*)**	**Class description**
A	0	22	0	21	Aspartic Peptidases
C	1	8	6	8	Cysteine Peptidases
I	4	2	2	1	Peptidase Inhibitors
M	12	9	17	6	Metallo Peptidases
S	39	19	27	21	Serine Peptidases
T	0	3	0	3	Threonine Peptidases
U	1	0	0	0	Peptidases of Unknown Catalytic Type

Another group of proteins thought to be important for promoting the growth of *A. cornea* is transporter proteins that support the transportation of growth-promoting molecules to *A. cornea*. Classification with Transporter Classification database (TCDB) revealed that the most prevalent type of transporters belonged to the ATP-binding cassette (ABC) superfamily with 42 of the proteins in this family found in the co-culture condition (Table 4; Supplementary Table S6). Another major family of transporters found in the co-culture was the fatty acid group translocation (FAT) family with 15 proteins found. These proteins are mostly predicted to locate in the plasma membrane, thus facilitating the transportation of proteins, lipids, carbohydrates, or secondary metabolites across the membrane. Interestingly, during the co-cultivation, *A. cornea* TBRC 12900 exhibited increased numbers of the putative vectorial glycosyl polymerization (VGP) family as well as auxiliary transport proteins.

**Table 4:** Number of transporters identified with TCDB.

**TCDB Class**	**Single culture (*P. mangrovi*)**	**Single culture (*A. cornea*)**	**Co-culture (*P. mangrovi*)**	**Co-culture (*A. cornea*)**	**Class description**
1	15	20	12	16	Channels/pores
2	24	14	18	14	Electrochemical potential-driven transporters
3	73	22	65	19	Primary active transporters
4	18	4	20	5	Group translocators
5	3	1	3	1	Transmembrane electron carriers
8	8	15	6	17	Accessory factors involved in transport
9	15	8	13	10	Incompletely characterized transport systems

**Figure 6:**
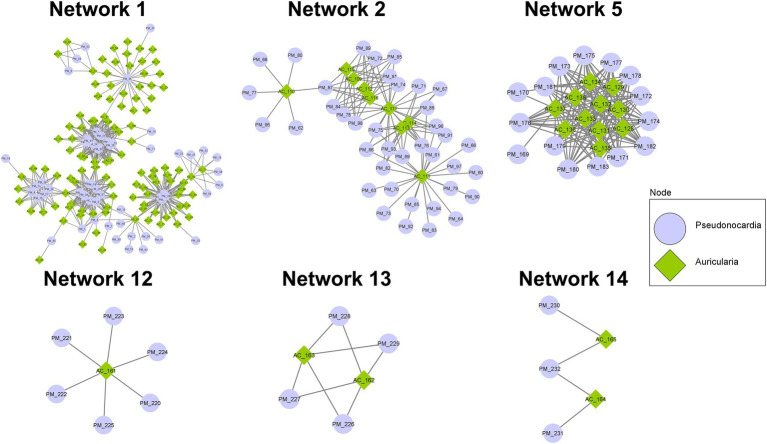
PPI networks showing possible interactions among protein domains identified from co-culture of *A. cornea* TBRC 12900 and *P. mangrovi* TBRC-BCC 42794. The 6 networks shown are those associated with BGCs known to be important for biosynthesis and transport of secondary metabolites, amino acids, polysaccharides, and lipids, as well as those involved in signal transduction. Protein domains of *A. cornea* origin are shown in green diamond and those of *P. mangrovi* origin are shown in blue circle.

With the notion that several proteins often function together concomitantly and sequentially to drive a metabolic pathway, proteins detected in the co-culture condition were analyzed using the STRING database and PPIDomainMiner to determine the protein–protein interaction (PPI) network *via* domain-domain interaction (DDI) pairs. With the proteins identified from the co-culture, a total of 15 PPI networks comprising at least 5 proteins and containing up to 167 proteins were found (Figure 6; Supplementary Table S7). In particular, major networks included 6 networks associated with BGCs that are known to be important for biosynthesis and transport of secondary metabolites, amino acids/peptides, saccharides, and lipids, as well as those involved in signal transduction (Network 1, Network 2, Network 5, Network12, Network 13, and Network 14). Among these networks, Network 1 contained the highest number of nodes (167) with 108 proteins of *A. cornea* origin and 59 proteins of *P. mangrovi* origin. Many proteins in this network can be mapped to BGCs involved in indole, *β*-lactone, and lanthipeptide synthesis pathways. Subcellular localization prediction by BUSCA revealed that many of these proteins are classified as plasma membrane proteins. In addition, several peptidases and glycosyl hydrolases important for the cleavage of peptide bonds in polypeptides/proteins and glycosidic bonds in carbohydrate substrates, respectively, were also found in this network. Network 2 constituted 48 proteins, most of which were mapped to NRPS, NRPS-like, and NAPAA biosynthetic gene clusters. BUSCA analysis showed that 29 of these proteins should be localized to plasma membranes and 1 protein from *P. mangrovi* is localized to extracellular space. Network 5 contained 26 proteins that can be classified by COG as those involved in secondary metabolites biosynthesis, transport, and catabolism. Among these proteins, 13 proteins are predicted as plasma membrane proteins. The main hub of interaction entailed a sulphite reductase protein involved in terpene synthesis pathway, which forms major interactions with cytochrome P450 domains. Network 12 contained 1 protein from *A. cornea* origin plus 6 proteins from *P. mangrovi* origin. All proteins are associated with CoA-transferase family III (Pfam family PF02515). In this network, all proteins from *P. mangrovi* origin are annotated as those involve in lipid transport and metabolism, especially the biosynthesis of nonribosomal lipopeptide iturin. Network 13 included 2 proteins from *A. cornea* origin and 4 proteins from *P. mangrovi* origin. They are thought to be NRPS-like enzymes related to shikimate dehydrogenase, which are implicated in the metabolism of aromatic amino acids and microansamycin synthesis pathways. Network 14 contained 2 proteins from *A. cornea* origin and 3 proteins from *P. mangrovi* origin that are associated with terpene biosynthesis.

Besides the networks mentioned above, several other PPI networks are identified to associate with transportation of secondary metabolites, peptides/amino acids, carbohydrates, or inorganic ions. For example, Network 3 comprised 46 proteins functioning as ABC-type transporters, all of which were predicted to localize in the plasma membrane by BUSCA. Network 6 contained 16 protein domains associated with transmembrane transport of various materials. Their functions are related to the major facilitator superfamily (MFS) transporter. Network 4 included 34 protein domains, most of which are members of the Na^+^ −transporting Carboxylic Acid Decarboxylase (NaT-DC) Family.

## Discussion

As the demand for mushrooms is increasing due to the rising trend of global food consumption, the improvement of mushroom productivity is important for socio-economic sustainability. The cultivable mushroom genus, *Auricularia*, is vastly consumed in Asian countries including Thailand either as food or traditional medicines. In general, *Auricularia* is cultivated in a substrate bag containing sawdust as the main component. In this study, we used the culture-independent approach and the next-generation sequencing technology to investigate the bacterial community in the spent mushroom substrate of *A. cornea* in order to find the growth-promoting bacteria for the cultivation of *A. cornea*. Consistent with the previous study in *A. cornea* and *Hypsizygus marmoreus* cultivation (Zhang et al., 2018; Sun et al., 2020), we found that the dominant bacterial phyla in the spent mushroom substrate of *A. cornea* were Firmicutes, Proteobacteria, Bacteroidetes, and Actinobacteria, respectively (Figure 1). Some studies on bacterial communities associated with mushroom cultivations showed different patterns of dominant bacterial phyla. For example, the dominant bacterial phyla in soil beneath black morel *Morchella sextelata* were Actinobacteria and Chloroflexi (Benucci et al., 2019). Proteobacteria and Chloroflexi were found dominantly in the casing soil of *Phlebopus porteutosus* (Yang et al., 2019), and the dominant bacterial phyla in *Agaricus* compost are changed during the cultivation processes (Kertesz and Thai, 2018). Several studies revealed that bacteria associated with mushrooms and mushroom substrates either had positive or negative effects on mushroom cultivation. Different roles of bacteria have been reported. Some bacteria can digest substrate to provide nutrients for mushrooms (Kertesz and Thai, 2018). Some bacteria have anti-fungal activities which inhibit the growth of other competitive or contaminated fungi, while some bacteria can produce specific enzymes to remove the inhibitor of primordial formation of mushrooms (Carrasco et al., 2018; Kertesz and Thai, 2018; Oh et al., 2018). Together with the result from our study, it is emphasized that different mushroom types and their cultivation processes require the augmentation of different bacterial communities to enhance mushroom growth.

We further investigated the bacterial species associated with *A. cornea* producing high yield (group A) and low yield (group B) of fruiting body and found that the alpha and beta diversities of the bacterial community in group A and group B were significantly different (Figure 2, Figure 3). Since the two groups of samples were divided based on fruiting body weights, these results demonstrate that bacterial community in the mushroom substrate is one of the factors influencing the mushroom production yield. Moreover, a set of differential bacterial species showing different abundance levels in group A and group B was identified (Figure 4). It was hypothesized that these bacterial species had a high potential to promote the growth of *A. cornea*. Co-cultivation experiment using the differential bacterial species was carried out to evaluate the effect exerted by these bacteria on mycelial growth. Figure 5 showed the different bacterial effects on mycelial growth in co-cultivation with *A. cornea*. *M. lotononidis* TBRC 5398 and *S. yanoikuyae* TBRC 5787 showed no enhancing effect on the growth of *A. cornea*. Remarkably, all three strains of *Pseudonocardia* significantly promoted the mycelial growth of *A. cornea*, with *P. mangrovi* TBRC-BCC 42794 showing the highest positive effect on the growth of *A. cornea*. Thus, these bacterial strains can be considered mushroom growth-promoting bacteria (MGPB) that enhance mushroom growth during cultivation.

Proteomic analyses revealed that proteins expressed during the co-cultivation of *A. cornea* TBRC 12900 and *P. mangrovi* TBRC-BCC 42794 may involve in many processes to aid in the growth of *A. cornea* by increasing nutrient availability (biofertilization) or the formation of growth-promoting factors (biostimulants) or suppressing diseases (bioprotectant). These proteins can be categorized into (a) enzymes involved in the degradation and modification of carbohydrates (hydrolases, glycosyltransferases, carbohydrate esterases, and carbohydrate-binding enzymes), (b) proteins responsible for cleavage of proteins/peptides (peptidases), (c) proteins predicted to be involved in intra- and extracellular transport (transporters), and (d) other proteins important for the formation of growth-promoting molecules or antimicrobial molecules. The increased numbers of *A. cornea*’s CAZy family proteins, proteins associated with biosynthetic pathways, and extracellular proteins in the co-culture condition compared to the single-culture conditions suggested there are interactions between *A. cornea* TBRC 12900 and *P. mangrovi* TBRC-BCC 42794. Specifically, the numbers of *A. cornea*’s CAZy family proteins that increased during the co-culture condition included auxiliary activity (AA) enzymes, carbohydrate esterases (CE), enzymes containing carbohydrate-binding module (CBM), and glycosyltransferases (GT). Even though the expression of CAZy enzymes could also be induced by components of the media (PDA supplemented with peptone) (Deka et al., 2011), the increase in several enzymes in the co-culture conditions most likely indicated that the expression certain *A. cornea*’s CAZy enzymes was chiefly induced by *P. mangrovi* and vice versa similarly to previous studies showing that co-cultivations of fungi with another fungi and fungi with bacteria induce or enhance the expression of CAZy enzyme (Juwaied et al., 2010; Noor El-Deen et al., 2014; Shahzadi et al., 2014; Moussa et al., 2019). Some compounds produced by bacteria might stimulate the secretion of mushroom CAZy enzyme. For example, cyclopeptide produced by *Bacillus cereus* BAC1 increases nitrogen source in mushroom substrate and enhances the secretion of amylase of *Pleurotus eryngii* (Chen et al., 2022). Moreover, it has been reported that gram-negative bacterial cell wall constituent lipopolysaccharide (LPS) stimulated the expression of fungal secondary metabolism (Khalil et al., 2014).

Especially during mycelial growth and fruiting body development, these enzymes should allow the mushroom to obtain nutrients from lignocellulosic substrates and maintain a diverse array of developmental processes, including energy metabolism, signaling, defense, cell wall structure formation, and carbohydrate-related post-translational modifications. These enzymes include AA9, and AA14 enzymes shown to be chitin-active families that are important for fungal cell wall remodeling during growth (Nagy et al., 2021). Their increased expression is consistent with chitin being one of the most important structural polymers of the fungal cell wall. AA3 is potentially bound to the fungal cell wall and may also aid in cell wall remodeling. GT2 belongs to chitin synthase, a key enzyme in the synthesis of chitin in fungal cell wall formation (Aspeborg et al., 2005). GH152 binds to glucan on fungal cell wall and possesses both *β*-1,3-glucanase and defense-related antimicrobial activities (Nagy et al., 2021). CE15 and GH43 enzymes possess ligninolytic or lignin-modifying activities (Hage and Rosso, 2021; Hao et al., 2022; Hua et al., 2022). CBM1-containing enzymes bind to crystalline cellulose and can thus involve in cellulose cleavage.

This study also found a high abundance of peptidases in the co-cultivation of *A. cornea* TBRC 12900 and *P. mangrovi* TBRC-BCC 42794, suggesting effective utilization of nitrogen resources and high protein content that can promote the growth of *A. cornea* TBRC 12900. Peptidases are a particular class of enzymes that play important roles in mycelial formation and elongation as they provide nitrogen and amino acids to the fungi or support cellular metabolism during growth (Liang et al., 2020). Interestingly, the numbers of cysteine- and metallo-peptidases of *P. mangrovi* increased significantly in the co-culture condition, compared to the single-culture conditions. The increase in the numbers of cysteine-proteases may help better protein turnover regulation and cellular homeostasis regulation (Dong et al., 2021). On the other hand, the increase in metallo-peptidases most likely helps in matrix remodeling, or microbial defense (Dong et al., 2021). Among the most abundant peptidases of *A. cornea*, the numbers of serine proteases markedly increased in the co-culture condition, compared to the single-culture conditions. This suggests an enhanced activity in protein digestion and processing, cell signaling, protein quality control, and intracellular protein turnover in the cell (Dong et al., 2021) associated with *A. cornea*’s mycelial growth. Among these serine proteases identified, the most abundant ones are S09 prolyl oligopeptidases, indicating increased ability to degrade proline-rich targets associated with protein maturation and extracellular degradation (Iwanicki et al., 2022). Another type of *A. cornea* proteases with a prominent increase in numbers during the co-culture condition is the Copia transposon peptidases, suggesting the increased activity of processing large Copia transposon transcripts into smaller products (Muszewska et al., 2011) during mycelial growth.

Another group of proteins that presents abundantly in the co-culture condition is transporters that transfer macromolecules including nutrients, metabolites, signaling molecules, and cellular constituents across the cell membrane. Most of these proteins are predicted to locate in the plasma membrane and they enable cells to acquire or remove compounds in and out of the cell. The high abundance of the ATP-binding cassette (ABC) superfamily and the fatty acid group translocation (FAT) family by the *P. mangrovi* TBRC-BCC 42794 in the co-culture condition should play a key role in exports of nutrients and growth-promoting substances to *A. cornea*. Interestingly, in the co-culture condition, the numbers of the putative vectorial glycosyl polymerization (VGP) family and the auxiliary transport proteins expressed by *A. cornea* TBRC 12900 were markedly elevated, compared to the single-culture conditions. The VGP family proteins usually act as polysaccharide synthase/exporters and may play a role in fungal deposition of polysaccharides (e.g., chitin) on the cell surface during fungal growth (Orlean and Funai, 2019).

When proteins identified in the co-culture condition were subjected to a prediction of the protein–protein interaction (PPI) network, the predicted 15 PPI networks, each containing 5–167 proteins, were shown to be associated with many cellular processes. These include networks (Network 1, 2, 5, 12, 13, and 14) associated with BGCs known to be important for biosynthesis and transport of secondary metabolites, amino acids, polysaccharides, and lipids, as well as those involved in signal transduction. For example, Network 1 shows the involvement of proteins in indole, *β*-lactone, and lanthipeptide biosynthesis pathways. Indoles have been shown to have broad biological activities including quorum sensing, spore formation, and antimicrobial resistance (Kim et al., 2017). For example, Indole-3-acetic acid (IAA) is the most abundant type of auxin that can be detected in the fruiting bodies and mycelia of mushrooms and has a positive impact on cell growth (Homer and Sperry, 2017), most likely by stimulating cell elongation and increase in cell wall synthesis (Mohite, 2013). Studies have shown that IAA is a signaling molecule in microorganisms, affecting gene expression in several microorganisms and affecting interactions between microorganisms (Fu et al., 2015). Additionally, indole compounds are found in mushroom’s fruiting bodies and are shown to be important for characteristic odors (Muszyńska et al., 2013; Homer and Sperry, 2017). These behaviors are consistent with the notion that the indole metabolism pathway in *A. cornea* will be highly active during mycelial growth and fruiting body development. Furthermore, other indoles, *β*-lactone, and lanthipeptide are bioactive metabolites that exhibit cytotoxic and antimicrobial properties (De Pascale et al., 2011; Homer and Sperry, 2017; Repka et al., 2017) and may be important for defense mechanisms against pathogens during growth. Network, 12 and 13 also underline biosynthetic pathways of bioactive compounds (*β*-lactone, iturin, and microansamycin) that confer antimicrobial properties. Network 2 of the predicted PPI contains proteins in the NRPS, NRPS-like, NAPAA biosynthetic gene clusters. Of particular interest is the identified heterobactin biosynthesis pathway that most likely aids in *A. cornea* growth. Heterobactin is a class of peptidic, extracellular siderophores, which are strong iron-binding molecules produced by many bacteria to acquire nutrient iron (Fe) from the environment (Wang et al., 2014). In many bacteria and fungi, the complex form of iron (Fe^3+^) from the environment is bound to siderophore and the Fe^3+^ siderophore is then moved across the cell membrane into the cell by energy-dependent systems. The Fe^3+^ ion is reduced into Fe^2+^ ion, which can then be utilized by the cell (Renshaw et al., 2002; Bhojiya et al., 2022; Singh et al., 2022). The production and secretion of siderophore will thus be beneficial for *A. cornea* to acquire, transport, and process the essential Fe ion during mushroom development and differentiation (Carrano et al., 2001; Bergeron et al., 2011). Proteins identified in Network 5 include sulphite reductase hemo protein involved in the synthesis of terpenes/terpenoids. Terpenes produced by fungi are volatile compounds that have diverse biological roles including mediating communication between fungi and bacteria and between fungi and animals, acting as toxins, repellents, or attractants to other organisms, as well as being considered building blocks for carotenoids synthesis (Quin et al., 2014; Schmidt et al., 2017; Jia et al., 2019). The terpenoid modifying enzymes, especially cytochrome P450 from *P. mangrovi* is also found in this network, reinforcing the notion of interaction between *A. cornea* TBRC 12900 and *P. mangrovi* TBRC-BCC 42794. Network 14 also contains protein interactions in the terpenoid synthesis pathway, most likely reiterating the interactions between the two organisms. In addition to networks devoted to biosynthesis pathways, several other networks focus on the transportation of molecules across the cell membrane. These include Network 3 emphasizing the ABC-transporter system and Network 6 emphasizing Major facilitator superfamily MFS transporter system. Consistent with their function as transporters, proteins in the networks possess transporter activity and are predicted to locate in plasma membranes. This supports the importance of transporter proteins for the transport and translocation of nutrients and metabolites to promote the growth of *A. cornea.* Some proteins cannot be specified in any PPI network, but may functionally support mycelial growth and fruiting body development. For example, two proteins expressed by *P. mangrovi* TBRC-BCC 42794 in the co-culture condition are those involved in ectoine biosynthesis pathway that most likely help the cell to cope with stresses encountered during growth. Ectoines provide cells with a considerable degree of tolerance against osmotic, temperature, and desiccation stresses (Pastor et al., 2010). Furthermore, under unfavorable conditions, ectoines are considered chemical chaperones by protecting the functionality of proteins, DNA, and macromolecular complexes, as well as stabilizing lipid bilayer (Richter et al., 2019).

In summary, this study showed that culture-independent metagenomic techniques can be employed to identify certain bacteria present in the mushroom substrate that exhibited mutualistic relationship and can promote the growth of *A. cornea* during cultivation. Based on the metagenomic data, some candidate strains from TBRC were then selected to evaluate their potential to promote the growth of *A. cornea*. Especially, *P. mangrovi* TBRC-BCC 42794 exhibited an ability as mushroom growth-promoting bacteria as the co-cultivation of *P. mangrovi* TBRC-BCC 42794 with *A. cornea* TBRC 12900 resulted in a high level of *A. cornea* mycelial growth. Proteomic analysis of proteins expressed during the co-culturing of *A. cornea* TBRC 12900 and *P. mangrovi* TBRC-BCC 42794 further revealed functions and probable interactions among the identified proteins. It is most likely that high mycelial growth in the co-culture condition results from concerted actions of certain proteins involved in binding, degradation, and modification of carbohydrates (hydrolases, glycosyltransferases, carbohydrate esterases, and carbohydrate-binding enzymes) including those important for cell wall generation/remodeling, peptidases including cysteine-, metallo-, and serine-peptidases, and transporters including the ABC-type transporter superfamily, the FAT transporter family, and the VGP family. Other proteins (e.g., metal-binding siderophores) can facilitate important cellular functions. Finally, proteins with roles in the formation of metabolites that can act as growth-promoting molecules (e.g., indoles, terpenes, and ectoines) as well as those normally contain antimicrobial activity (e.g., *β*-lactones, lanthipeptides, and iturins) important for defense mechanisms against pathogen also most likely promote of *A. cornea* mycelial growth and fruiting body development. The work showed that a combination of metagenomics, proteomics, and functional annotation analysis can lead to the identification of important mushroom growth-promoting bacteria and elucidation of proposed protein players involved in enhancing mycelial growth of *A. cornea*.

## Data availability statement

The datasets presented in this study can be found in online repositories. The names of the repository/repositories and accession number(s) can be found at: NCBI BioProject, PRJNA862630; ProteomeXchange, PXD035814.

## Author contributions

SM, PS, CP, LE, and SI conceived and designed research. SM and SW coordinated with the farm operator. CP, SK, and SM conducted experiments. PS, PH, and SI performed bioinformatic analysis. CP, PH, and SI wrote the manuscript. All authors contributed to the article and approved the submitted version.

## Funding

This work was supported by National Science and Technology Development Agency.

## Conflict of interest

The authors declare that the research was conducted in the absence of any commercial or financial relationships that could be construed as a potential conflict of interest.

## Publisher’s note

All claims expressed in this article are solely those of the authors and do not necessarily represent those of their affiliated organizations, or those of the publisher, the editors and the reviewers. Any product that may be evaluated in this article, or claim that may be made by its manufacturer, is not guaranteed or endorsed by the publisher.

## Footnotes

^1^ http://www.cazy.org/

^2^ http://cys.bios.niu.edu/dbCAN2

^3^ https://www.ebi.ac.uk/merops/

^4^ http://tcdb.org/

^5^ http://www.ncbi.nlm.nih.gov/COG

^6^ http://busca.biocomp.unibo.it

^7^ http://ppidm.loria.fr/

^8^ https://string-db.org/
