# Supplementary material for: Beneficial bacterial-Auricularia cornea interactions fostering growth enhancement identified from microbiota present in spent mushroom substrate
Source: Front Microbiol. 2022 Oct 10;13:1006446. doi: 10.3389/fmicb.2022.1006446 (PMC9589457; doi:10.3389/fmicb.2022.1006446)
Supplement: Supplementary file 2 [file Data_Sheet_1.docx]

***Supplementary Material***

Beneficial Bacterial-*Auricularia cornea* Interactions Fostering Growth Enhancement Identified from Microbiota Present in Spent Mushroom Substrate

**Chitwadee Phithakrotchanakoon^1^, Sermsiri Mayteeworakoon^1^, Paopit Siriarchawatana^1^, Supattra Kitikhun^1^, Piyanun Harnpicharnchai^1^, Supaporn Wansom^2^, Lily Eurwilaichitr^2^, Supawadee Ingsriswang^1^***

^1^Microbial Systems and Computational Biology Research Team, Thailand Bioresource Research Center, National Center for Genetic Engineering and Biotechnology, National Science and Technology Development Agency, Pathum Thani, Thailand

^2^National Energy Technology Center, National Science and Technology Development Agency, Pathum Thani, Thailand

*** Correspondence:**Supawadee Ingsriswang
supawadee@biotec.or.th


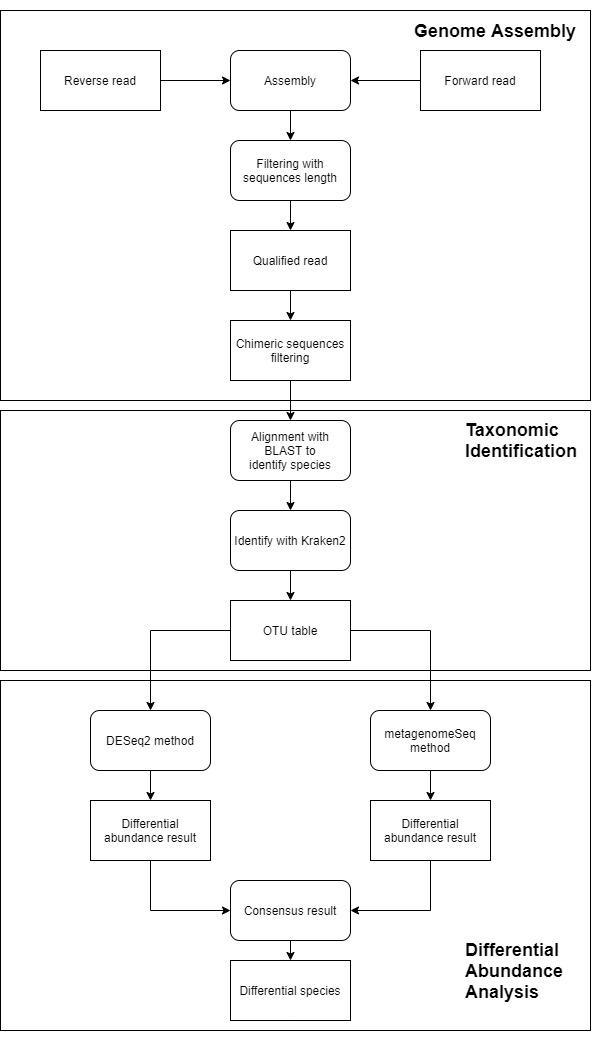


# Figure S1 Workflow for differential species identification

# Table S1 Total weight of the fruiting bodies from each sample in group A and group B

| **Group** | **Sample name** | **Total weight of fruiting body (g) per kilogram of substrate** |
| --- | --- | --- |
| High yield | A1 | 220 |
| High yield | A2 | 206 |
| High yield | A3 | 232 |
| High yield | A4 | 258 |
| High yield | A5 | 234 |
| Low Yield | B1 | 130 |
| Low Yield | B2 | 135 |
| Low Yield | B3 | 139 |
| Low Yield | B4 | 101 |
| Low Yield | B5 | 131 |

**Table S2** Sequencing data and diversity indices of each sample

| **Sample name** | **No. of raw reads** | **No. of qualified reads** | **No. of nonchimeric reads** | **No. of OTUs at species level** | **Chao 1** | **Shannon** | **Simpson** |
| --- | --- | --- | --- | --- | --- | --- | --- |
| A1 | 97,632 | 96,529 | 71,407 | 740 | 762.5238 | 4.541739 | 0.965346 |
| A2 | 106,734 | 105,432 | 77,764 | 722 | 756.7059 | 4.285603 | 0.963174 |
| A3 | 104,356 | 103,173 | 84,315 | 742 | 759.5000 | 4.677596 | 0.977219 |
| A4 | 91,316 | 90,305 | 72,575 | 730 | 737.8750 | 4.673246 | 0.974298 |
| A5 | 100,339 | 98,914 | 80,755 | 677 | 711.4375 | 4.45513 | 0.973936 |
| B1 | 98,802 | 97,610 | 76,879 | 641 | 681.7143 | 3.856913 | 0.942056 |
| B2 | 89,367 | 88,117 | 67,794 | 670 | 730.0600 | 4.218386 | 0.962574 |
| B3 | 92,999 | 91,806 | 68,978 | 561 | 656.0000 | 3.651061 | 0.937663 |
| B4 | 95,592 | 94,391 | 73,993 | 664 | 701.8182 | 4.223615 | 0.954274 |
| B5 | 99,978 | 98,906 | 81,062 | 601 | 688.2083 | 3.552377 | 0.911927 |


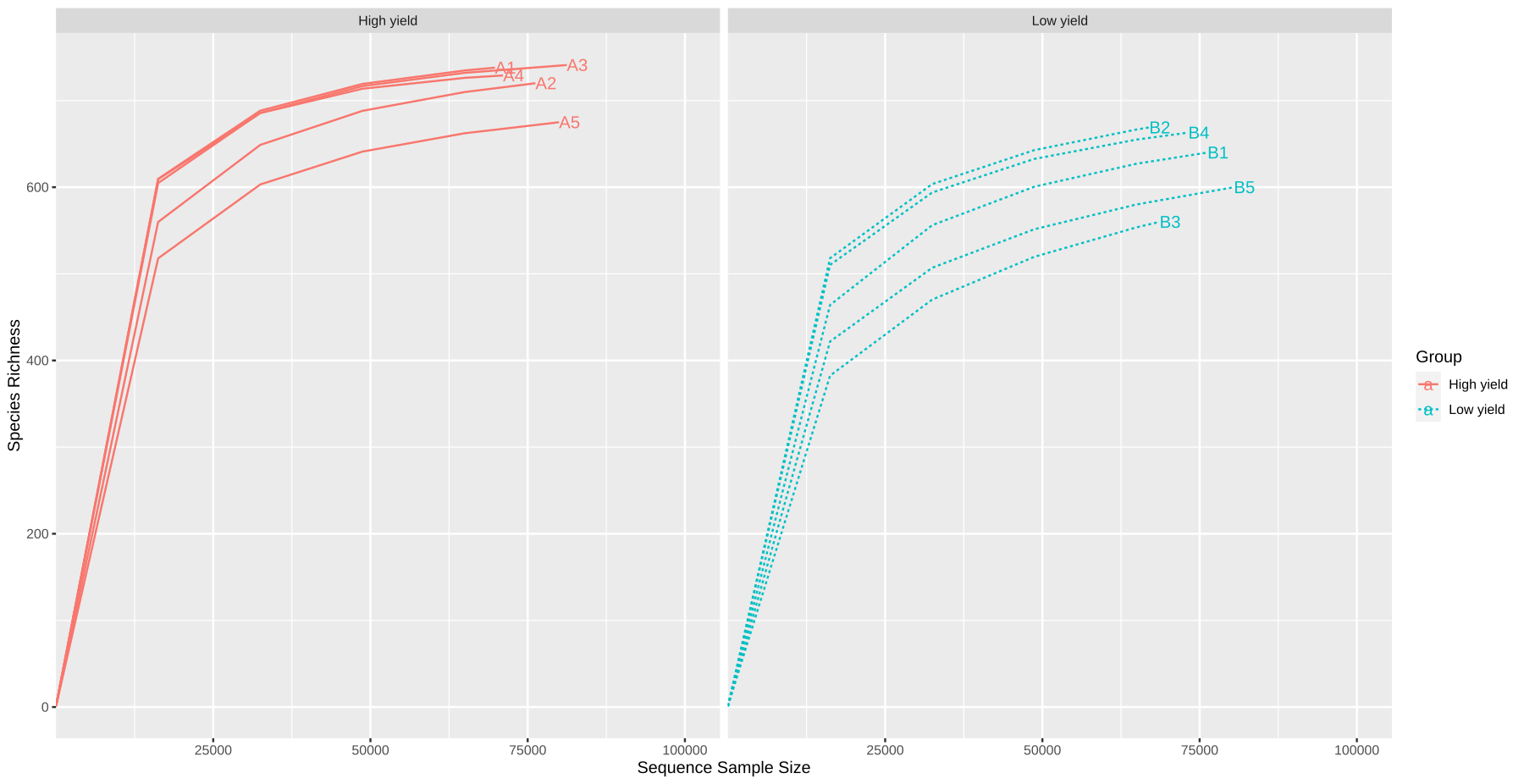


# Figure S2 Rarefaction curves of observed operational taxonomic units (OTUs)

# Table S3 Species with differential abundance between group A and group B

# Result from DESeq2 analysis (p-Values < 0.05)

| **Species** | **log2FoldChange** | **p-Values** |
| --- | --- | --- |
| *Paracoccus pantotrophus* | -8.644258592 | 3.67E-11 |
| *Microvirga massiliensis* | -7.485055899 | 5.78E-06 |
| *Pseudonocardia mangrovi* | -6.34849546 | 1.23E-05 |
| *Bacillus fortis* | -6.666694342 | 3.57E-05 |
| *Bacillus maritimus* | -5.136296782 | 0.000268013 |
| *Sphingobium jiangsuense* | -6.598297557 | 0.000268013 |
| *Thauera humireducens* | -8.14429647 | 0.000574765 |
| *Luteimonas composti* | -8.307054351 | 0.000964308 |
| *Rhodobacter blasticus* | -4.657950741 | 0.003508247 |
| *Chelatococcus caeni* | -5.45368533 | 0.007005525 |
| *Pseudonocardia spinosa* | -5.23312447 | 0.008290676 |
| *Gemmobacter nanjingensis* | -4.766983882 | 0.008944583 |
| *Bacillus sediminis* | -6.908081723 | 0.012139246 |
| *Schlegelella thermodepolymerans* | -5.099701987 | 0.012139246 |
| *Aquamicrobium defluvii* | -5.376445992 | 0.012630101 |
| *Aquamicrobium aestuarii* | -5.110516294 | 0.014065454 |
| *Conexibacter woesei* | -5.190339213 | 0.022584996 |
| *Corallococcus macrosporus* | 2.9759225 | 0.0238914 |
| *Phenylobacterium panacis* | -6.499437503 | 0.02480062 |
| *Pseudofulvimonas gallinarii* | -7.386850689 | 0.026127039 |
| *Paracoccus solventivorans* | -4.99360555 | 0.029463457 |
| *Parapusillimonas granuli* | -6.80748401 | 0.029463457 |
| *Bacillus lycopersici* | 1.669696192 | 0.032627873 |
| *Oxalicibacterium faecigallinarum* | -2.203321738 | 0.03440316 |
| *Promicromonospora citrea* | -3.195400449 | 0.03440316 |
| *Glaciimonas immobilis* | -2.235056358 | 0.035868487 |
| *Leucobacter margaritiformis* | -6.348954893 | 0.040623532 |
| *Bacillus kexueae* | 1.949115263 | 0.0429266 |
| *Hyphomicrobium nitrativorans* | -3.717225304 | 0.0429266 |
| *Pedomicrobium manganicum* | -6.68845196 | 0.0429266 |
| *Chelatococcus composti* | -4.428365969 | 0.043326354 |
| *Oceanobacillus chironomi* | 1.794740322 | 0.04545468 |
| *Phyllobacterium catacumbae* | -2.605833207 | 0.04545468 |
| *Pseudolabrys taiwanensis* | -3.680643568 | 0.04545468 |

1. Result from metagenomeSeq analysis (p-Values < 0.05)

| **Species** | **log2FoldChange** | **p-Values** |
| --- | --- | --- |
| *Luteimonas composti* | -6.25741111 | 0.000278186 |
| *Nitrosomonas halophila* | -5.896219322 | 0.001826531 |
| *Paracoccus pantotrophus* | -6.563995903 | 0.003699099 |
| *Pseudonocardia mangrovi* | -4.300296984 | 0.005911526 |
| *Sphingobium jiangsuense* | -4.719121336 | 0.005911526 |
| *Bacillus sediminis* | -4.347411377 | 0.008185579 |
| *Microvirga massiliensis* | -4.870451121 | 0.008185579 |
| *Parapusillimonas granuli* | -4.501394929 | 0.008185579 |
| *Phenylobacterium panacis* | -4.554855929 | 0.008185579 |
| *Pseudofulvimonas gallinarii* | -4.755018282 | 0.008185579 |
| *Comamonas zonglianii* | -3.726798551 | 0.008505619 |
| *Rhodobacter blasticus* | -3.053618389 | 0.011315259 |
| *Hyphomicrobium nitrativorans* | -2.904703267 | 0.013040058 |
| *Leucobacter margaritiformis* | -4.121236096 | 0.013040058 |
| *Phyllobacterium catacumbae* | -2.225028229 | 0.014991617 |
| *Candidimonas bauzanensis* | -3.827069431 | 0.015193482 |
| *Pseudonocardia spinosa* | -3.549541831 | 0.015193482 |
| *Chelatococcus caeni* | -3.563363505 | 0.015402926 |
| *Pedomicrobium manganicum* | -4.161077656 | 0.015402926 |
| *Bacillus maritimus* | -3.396136857 | 0.025444989 |
| *Bacillus fortis* | -4.0776516 | 0.026193513 |
| *Promicromonospora citrea* | -2.224273116 | 0.026351119 |
| *Schlegelella thermodepolymerans* | -3.362827499 | 0.026351119 |
| *Thauera humireducens* | -4.827572882 | 0.026351119 |
| *Oxalicibacterium faecigallinarum* | -2.357397533 | 0.035606445 |
| *Aquamicrobium aestuarii* | -3.263882593 | 0.04475703 |
| *Glaciimonas immobilis* | -2.267848023 | 0.04475703 |
| *Elioraea tepidiphila* | -3.49348526 | 0.045511713 |
| *Caldimonas hydrothermale* | -1.884682281 | 0.045937845 |

1. Consensus-based result from DESeq2 and metagenomeSeq analysis

| **Species** | **p-Value** | |
| --- | --- | --- |
|  | **DESeq2** | **metagenomeSeq** |
| *Luteimonas composti* | 0.000964308 | 0.000278186 |
| *Paracoccus pantotrophus* | 0 | 0.003699099 |
| *Sphingobium jiangsuense* | 0.000268013 | 0.005911526 |
| *Pseudonocardia mangrovi* | 0.0000123 | 0.005911526 |
| *Microvirga massiliensis* | 0.00000578 | 0.008185579 |
| *Parapusillimonas granuli* | 0.029463457 | 0.008185579 |
| *Pseudofulvimonas gallinarii* | 0.026127039 | 0.008185579 |
| *Phenylobacterium panacis* | 0.02480062 | 0.008185579 |
| *Bacillus sediminis* | 0.012139246 | 0.008185579 |
| *Rhodobacter blasticus* | 0.003508247 | 0.011315259 |
| *Hyphomicrobium nitrativorans* | 0.0429266 | 0.013040058 |
| *Leucobacter margaritiformis* | 0.040623532 | 0.013040058 |
| *Phyllobacterium catacumbae* | 0.04545468 | 0.014991617 |
| *Pseudonocardia spinosa* | 0.008290676 | 0.015193482 |
| *Pedomicrobium manganicum* | 0.0429266 | 0.015402926 |
| *Chelatococcus caeni* | 0.007005525 | 0.015402926 |
| *Bacillus maritimus* | 0.000268013 | 0.025444989 |
| *Bacillus fortis* | 0.0000357 | 0.026193513 |
| *Promicromonospora citrea* | 0.03440316 | 0.026351119 |
| *Schlegelella thermodepolymerans* | 0.012139246 | 0.026351119 |
| *Thauera humireducens* | 0.000574765 | 0.026351119 |
| *Oxalicibacterium faecigallinarum* | 0.03440316 | 0.035606445 |
| *Glaciimonas immobilis* | 0.035868487 | 0.04475703 |
| *Aquamicrobium aestuarii* | 0.014065454 | 0.04475703 |

**Table S4** Families of CAZymes identified from the single- and co-cultures

| **CAZymes family** | **Single culture (*P. mangrovi*)** | **Single culture (*A. cornea*)** | **Co-culture (*P. mangrovi*)** | **Co-culture (*A. cornea*)** |
| --- | --- | --- | --- | --- |
| AA14 | 0 | 0 | 0 | 2 |
| AA2 | 1 | 0 | 0 | 0 |
| AA3 | 0 | 0 | 0 | 2 |
| AA7 | 1 | 0 | 4 | 0 |
| AA9 | 0 | 0 | 0 | 1 |
| CBM1 | 0 | 0 | 0 | 2 |
| CBM13 | 3 | 0 | 0 | 0 |
| CBM2 | 0 | 1 | 0 | 1 |
| CBM42 | 0 | 0 | 0 | 1 |
| CBM5 | 0 | 1 | 0 | 0 |
| CBM50 | 0 | 1 | 0 | 0 |
| CE10 | 1 | 2 | 0 | 2 |
| CE14 | 1 | 0 | 1 | 0 |
| CE15 | 1 | 0 | 1 | 1 |
| CE16 | 0 | 0 | 0 | 1 |
| CE4 | 1 | 0 | 3 | 0 |
| GH0 | 1 | 0 | 1 | 0 |
| GH10 | 0 | 1 | 0 | 0 |
| GH105 | 0 | 1 | 0 | 1 |
| GH13 | 2 | 0 | 1 | 0 |
| GH15 | 2 | 0 | 1 | 0 |
| GH152 | 0 | 0 | 0 | 1 |
| GH16 | 0 | 1 | 0 | 1 |
| GH18 | 0 | 1 | 0 | 1 |
| GH2 | 0 | 0 | 2 | 1 |
| GH23 | 1 | 0 | 2 | 0 |
| GH28 | 1 | 0 | 0 | 1 |
| GH29 | 0 | 1 | 0 | 1 |
| GH3 | 1 | 0 | 1 | 0 |
| GH38 | 0 | 1 | 0 | 2 |
| GH4 | 1 | 0 | 1 | 0 |
| GH43 | 0 | 1 | 0 | 2 |
| GH5 | 0 | 4 | 0 | 1 |
| GH6 | 3 | 0 | 2 | 0 |
| GH76 | 0 | 0 | 1 | 0 |
| GH79 | 0 | 2 | 0 | 1 |
| GT0 | 1 | 0 | 1 | 0 |
| GT1 | 1 | 1 | 1 | 0 |
| GT2 | 14 | 2 | 13 | 5 |
| GT20 | 1 | 0 | 1 | 1 |
| GT26 | 1 | 0 | 1 | 0 |
| GT28 | 1 | 0 | 2 | 0 |
| GT3 | 0 | 1 | 0 | 0 |
| GT35 | 0 | 0 | 0 | 1 |
| GT39 | 0 | 0 | 0 | 1 |
| GT4 | 4 | 0 | 5 | 0 |
| GT41 | 0 | 1 | 0 | 0 |
| GT48 | 0 | 1 | 0 | 1 |
| GT51 | 3 | 0 | 2 | 0 |
| GT83 | 1 | 0 | 1 | 0 |
| GT87 | 1 | 0 | 1 | 0 |
| GT9 | 1 | 0 | 1 | 0 |
| GT90 | 0 | 1 | 0 | 1 |
| PL0 | 0 | 1 | 0 | 1 |
| PL14 | 0 | 1 | 0 | 0 |

**Table S5** Families of peptidases identified by MEROPS from the single- and co-culture conditions

| **MEROPS peptidase family** | **Single culture (*P. mangrovi*)** | **Single culture (*A. cornea*)** | **Co-culture (*P. mangrovi*)** | **Co-culture (*A. cornea*)** |
| --- | --- | --- | --- | --- |
| A1 | 0 | 1 | 0 | 2 |
| A11 | 0 | 8 | 0 | 10 |
| A2 | 0 | 5 | 0 | 5 |
| A28 | 0 | 7 | 0 | 3 |
| A31 | 0 | 1 | 0 | 1 |
| C110 | 0 | 0 | 1 | 0 |
| C12 | 0 | 0 | 0 | 1 |
| C14 | 0 | 1 | 0 | 0 |
| C19 | 0 | 1 | 0 | 0 |
| C26 | 0 | 0 | 1 | 0 |
| C40 | 0 | 0 | 1 | 0 |
| C44 | 1 | 0 | 2 | 0 |
| C48 | 0 | 6 | 0 | 7 |
| C56 | 0 | 0 | 1 | 0 |
| I4 | 2 | 1 | 1 | 0 |
| I51 | 0 | 0 | 0 | 1 |
| I87 | 2 | 1 | 1 | 0 |
| M1 | 0 | 0 | 2 | 0 |
| M12 | 0 | 0 | 0 | 1 |
| M16 | 1 | 1 | 0 | 0 |
| M17 | 2 | 0 | 1 | 0 |
| M20 | 3 | 0 | 4 | 0 |
| M23 | 1 | 1 | 0 | 0 |
| M24 | 0 | 2 | 0 | 0 |
| M28 | 0 | 0 | 1 | 1 |
| M3 | 1 | 0 | 1 | 0 |
| M35 | 0 | 1 | 0 | 1 |
| M36 | 0 | 1 | 0 | 0 |
| M38 | 3 | 1 | 5 | 1 |
| M41 | 1 | 1 | 1 | 1 |
| M50 | 0 | 0 | 2 | 0 |
| M67 | 0 | 1 | 0 | 1 |
| S1 | 0 | 0 | 2 | 1 |
| S11 | 0 | 0 | 1 | 0 |
| S12 | 2 | 0 | 2 | 1 |
| S16 | 4 | 0 | 2 | 1 |
| S33 | 12 | 5 | 5 | 2 |
| S41 | 0 | 1 | 1 | 0 |
| S45 | 1 | 0 | 1 | 0 |
| S49 | 10 | 0 | 5 | 0 |
| S8 | 3 | 4 | 2 | 6 |
| S9 | 7 | 9 | 6 | 10 |
| T1 | 0 | 1 | 0 | 0 |
| T2 | 0 | 1 | 0 | 0 |
| T3 | 0 | 1 | 0 | 3 |
| U72 | 1 | 0 | 0 | 0 |

**Table S6** TC Families identified from the single- and co-cultures

| **TC family** | **Single culture (*P. mangrovi*)** | **Single culture (*A. cornea*)** | **Co-culture (*P. mangrovi*)** | **Co-culture (*A. cornea*)** |
| --- | --- | --- | --- | --- |
| 1.A.1 | 1 | 0 | 1 | 0 |
| 1.A.112 | 0 | 0 | 1 | 0 |
| 1.A.115 | 5 | 2 | 3 | 1 |
| 1.A.17 | 0 | 0 | 0 | 1 |
| 1.A.23 | 0 | 1 | 0 | 0 |
| 1.A.33 | 1 | 0 | 1 | 0 |
| 1.A.35 | 1 | 0 | 0 | 0 |
| 1.A.7 | 0 | 0 | 0 | 1 |
| 1.A.87 | 1 | 2 | 1 | 2 |
| 1.A.9 | 0 | 1 | 0 | 0 |
| 1.C.104 | 0 | 0 | 0 | 1 |
| 1.E.40 | 1 | 0 | 1 | 0 |
| 1.F.2 | 0 | 2 | 0 | 1 |
| 1.F.3 | 0 | 1 | 0 | 0 |
| 1.H.3 | 1 | 0 | 0 | 0 |
| 1.I.1 | 3 | 9 | 2 | 3 |
| 1.I.3 | 0 | 1 | 0 | 1 |
| 1.N.7 | 0 | 0 | 0 | 1 |
| 1.P.1 | 0 | 1 | 0 | 1 |
| 1.R.1 | 0 | 0 | 0 | 3 |
| 1.W.6 | 1 | 0 | 2 | 0 |
| 2.A.1 | 10 | 5 | 8 | 6 |
| 2.A.103 | 0 | 0 | 1 | 0 |
| 2.A.105 | 0 | 1 | 0 | 1 |
| 2.A.108 | 1 | 0 | 0 | 0 |
| 2.A.14 | 1 | 0 | 1 | 0 |
| 2.A.16 | 0 | 0 | 0 | 1 |
| 2.A.20 | 1 | 0 | 0 | 0 |
| 2.A.21 | 1 | 1 | 1 | 1 |
| 2.A.25 | 0 | 0 | 1 | 0 |
| 2.A.28 | 1 | 0 | 1 | 0 |
| 2.A.29 | 0 | 2 | 0 | 0 |
| 2.A.3 | 2 | 1 | 1 | 2 |
| 2.A.36 | 0 | 0 | 0 | 1 |
| 2.A.40 | 1 | 0 | 0 | 0 |
| 2.A.43 | 0 | 1 | 0 | 1 |
| 2.A.53 | 1 | 0 | 1 | 0 |
| 2.A.57 | 0 | 2 | 0 | 1 |
| 2.A.59 | 0 | 0 | 1 | 0 |
| 2.A.63 | 1 | 0 | 0 | 0 |
| 2.A.7 | 1 | 1 | 0 | 0 |
| 2.A.8 | 2 | 0 | 2 | 0 |
| 2.A.80 | 1 | 0 | 0 | 0 |
| 3.A.1 | 43 | 7 | 42 | 5 |
| 3.A.11 | 3 | 0 | 3 | 0 |
| 3.A.12 | 1 | 0 | 0 | 0 |
| 3.A.16 | 0 | 2 | 0 | 1 |
| 3.A.18 | 0 | 2 | 0 | 1 |
| 3.A.2 | 0 | 0 | 1 | 1 |
| 3.A.20 | 0 | 0 | 0 | 1 |
| 3.A.23 | 2 | 0 | 2 | 0 |
| 3.A.24 | 2 | 1 | 1 | 1 |
| 3.A.25 | 0 | 2 | 0 | 0 |
| 3.A.29 | 1 | 0 | 1 | 0 |
| 3.A.3 | 3 | 1 | 4 | 1 |
| 3.A.30 | 0 | 1 | 0 | 0 |
| 3.A.31 | 0 | 1 | 0 | 3 |
| 3.A.4 | 1 | 0 | 1 | 0 |
| 3.A.5 | 2 | 1 | 1 | 1 |
| 3.A.7 | 1 | 1 | 1 | 0 |
| 3.A.8 | 0 | 1 | 0 | 1 |
| 3.A.9 | 6 | 0 | 3 | 0 |
| 3.B.1 | 0 | 0 | 1 | 1 |
| 3.D.1 | 5 | 2 | 1 | 2 |
| 3.D.7 | 3 | 0 | 3 | 0 |
| 4.A.1 | 1 | 0 | 0 | 0 |
| 4.A.6 | 0 | 0 | 2 | 0 |
| 4.C.1 | 15 | 1 | 15 | 0 |
| 4.C.2 | 0 | 1 | 0 | 0 |
| 4.C.3 | 1 | 0 | 1 | 0 |
| 4.D.1 | 0 | 2 | 1 | 5 |
| 4.H.1 | 1 | 0 | 1 | 0 |
| 5.A.3 | 3 | 0 | 2 | 0 |
| 5.B.1 | 0 | 1 | 1 | 1 |
| 8.A.104 | 1 | 3 | 1 | 4 |
| 8.A.111 | 0 | 1 | 0 | 0 |
| 8.A.114 | 0 | 0 | 0 | 1 |
| 8.A.117 | 1 | 0 | 1 | 0 |
| 8.A.126 | 0 | 1 | 0 | 1 |
| 8.A.173 | 0 | 2 | 0 | 1 |
| 8.A.188 | 0 | 0 | 0 | 1 |
| 8.A.192 | 0 | 1 | 0 | 0 |
| 8.A.21 | 2 | 0 | 1 | 0 |
| 8.A.23 | 0 | 1 | 0 | 1 |
| 8.A.32 | 0 | 1 | 0 | 2 |
| 8.A.34 | 0 | 1 | 0 | 1 |
| 8.A.5 | 1 | 2 | 1 | 2 |
| 8.A.51 | 2 | 0 | 1 | 0 |
| 8.A.67 | 0 | 1 | 0 | 1 |
| 8.A.85 | 1 | 0 | 1 | 0 |
| 8.A.92 | 0 | 1 | 0 | 2 |
| 9.A.15 | 0 | 1 | 0 | 1 |
| 9.A.19 | 0 | 1 | 0 | 0 |
| 9.A.29 | 0 | 0 | 0 | 1 |
| 9.A.3 | 0 | 1 | 0 | 1 |
| 9.A.34 | 0 | 1 | 0 | 0 |
| 9.B.111 | 1 | 0 | 0 | 0 |
| 9.B.119 | 0 | 1 | 0 | 1 |
| 9.B.14 | 1 | 0 | 1 | 0 |
| 9.B.142 | 1 | 0 | 1 | 1 |
| 9.B.18 | 1 | 0 | 1 | 0 |
| 9.B.191 | 1 | 1 | 1 | 1 |
| 9.B.259 | 1 | 0 | 1 | 0 |
| 9.B.27 | 1 | 0 | 0 | 0 |
| 9.B.278 | 0 | 0 | 0 | 2 |
| 9.B.282 | 0 | 0 | 1 | 0 |
| 9.B.29 | 0 | 0 | 1 | 0 |
| 9.B.302 | 1 | 0 | 0 | 0 |
| 9.B.33 | 1 | 0 | 0 | 0 |
| 9.B.34 | 3 | 0 | 3 | 0 |
| 9.B.35 | 0 | 1 | 0 | 0 |
| 9.B.36 | 1 | 0 | 1 | 0 |
| 9.B.392 | 0 | 1 | 0 | 0 |
| 9.B.416 | 0 | 0 | 0 | 1 |
| 9.B.45 | 0 | 0 | 0 | 1 |
| 9.B.62 | 1 | 0 | 1 | 0 |
| 9.B.97 | 1 | 0 | 1 | 0 |
